# Deep Earth forcing and paleoclimate evolution

**DOI:** 10.1093/nsr/nwag502

**Published:** 2026-08-19

**Authors:** Xinwei Liu, Qiuming Cheng

**Affiliations:** School of Earth Sciences and Engineering, Sun Yat–sen University, China; School of Earth Sciences and Engineering, Sun Yat–sen University, China; School of Earth Sciences and Resources, China University of Geosciences (Beijing), China; State Key Laboratory of Geological Processes and Mineral Resources, China University of Geosciences (Beijing), China; DDE Frontier Science Center, China University of Geosciences (Beijing), China

Reconstructing Earth’s climate evolution and identifying the mechanisms governing long-term environmental change remain central objectives of paleoclimate research. Understanding how climate systems responded to past perturbations provides critical context for interpreting present climate dynamics and future trajectories. Increasingly, the field has shifted from empirical interpretations of geological archives toward process-based explanations grounded in Earth system dynamics.

Despite substantial progress, a fundamental question remains unresolved: what ultimately controls climate evolution over geological timescales? Existing explanations are commonly framed as a dichotomy. One perspective treats deep geological processes as external boundary conditions, emphasizing unidirectional forcing through volcanic outgassing and volatile fluxes [1]. Another emphasizes internal feedbacks within surface reservoirs, including the atmosphere, ocean, and biosphere, while simplifying processes within the solid Earth. However, this separation is challenged by persistent observations in Earth history. Previous studies have revealed important links among tectonics, carbon cycling, weathering, and paleogeography, yet an integrated framework connecting deep forcing with climate evolution remains lacking [2]. For example, geologically comparable deep events can produce markedly different climatic outcomes, whereas relatively modest perturbations may occasionally trigger major environmental transitions. Here we synthesize the coupling mechanisms linking deep Earth processes and surface climate evolution and propose a conceptual framework in which climate dynamics are governed by the interaction among deep forcing, surface modulation, and feedback processes (Fig. 1). Within this framework, the climatic consequences of deep events depend not simply on forcing magnitude but on how surface reservoirs redistribute, amplify or suppress perturbations and on how feedback processes reorganize system behavior through time. Climate states and transitions emerge from changing interactions across the Earth system rather than from isolated forcing mechanisms.

**Figure 1.**
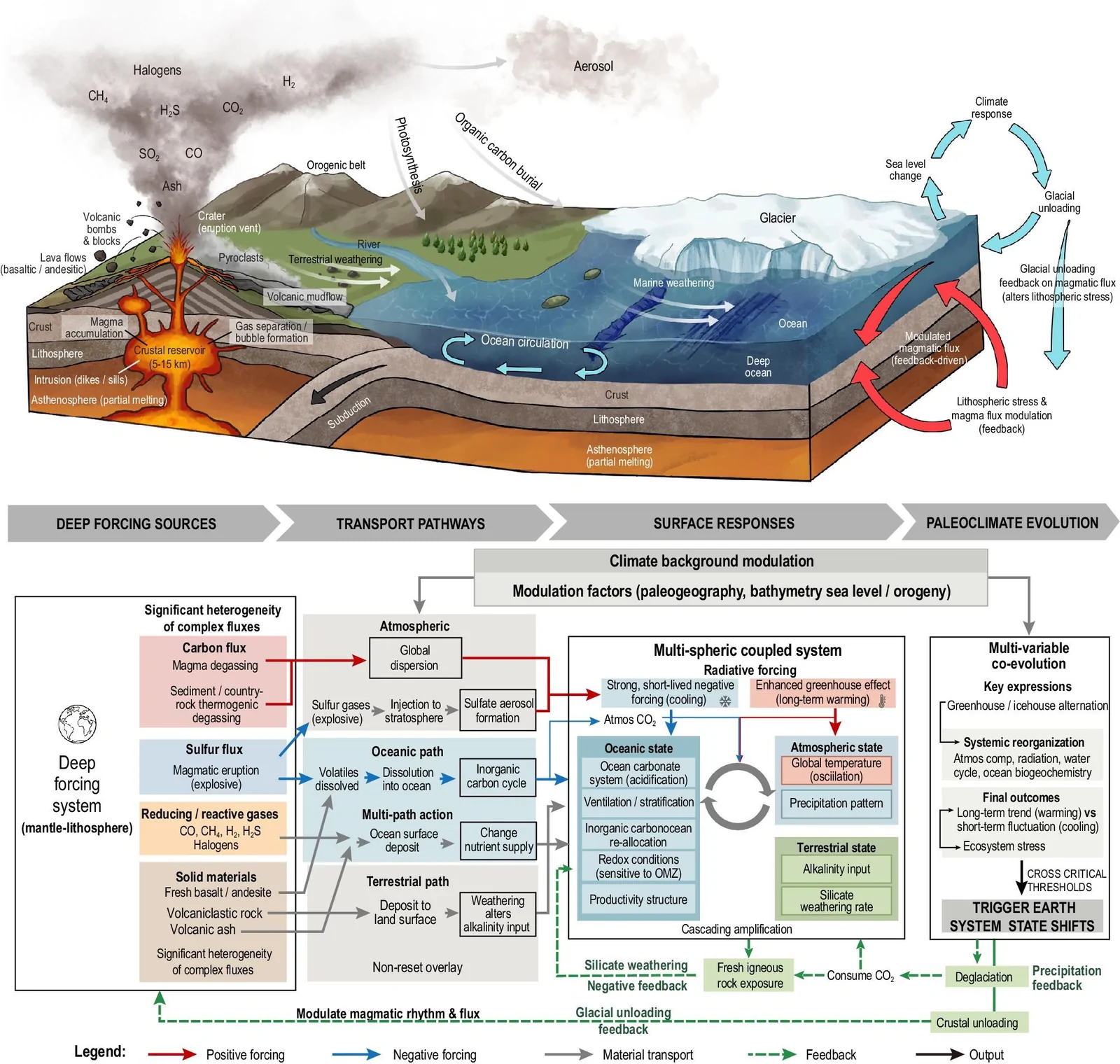
Upper panel: deep Earth forcing and surface multi-sphere interaction model. Lower panel: schematic illustration of the multi-pathway transmission and system coupling mechanisms linking deep forcing sources to paleoclimate evolution.

In this framework, deep forcing refers to geological inputs from Earth’s interior, including carbon, sulfur, halogens, volcanic ash, and lithological changes. Surface modulation represents the state-dependent regulation of these perturbations by surface reservoirs, whereas feedback processes include internal mechanisms that amplify or stabilize climate responses, such as weathering and carbon cycling [3]. Deep Earth forcing is intrinsically multi-component. Geological processes associated with mantle dynamics, magmatism and tectonically mediated remobilization of crustal reservoirs introduce multiple fluxes into the exogenic system simultaneously. These fluxes include carbon gas, sulfur species, halogens, reduced gases, volcanic ash and freshly exposed lithologies. Their climatic effects depend not only on the magnitude of individual fluxes but also their relative duration and interaction.

Carbon input illustrates this complexity [4]. A fundamental distinction exists between juvenile mantle-derived carbon and crustal-recycled carbon [5]. Mantle degassing supplies a long-term carbon source to the exogenic system, whereas thermal decomposition of sedimentary organic matter and carbonate reservoirs can generate substantially large transient releases of previously stored carbon [6]. These sources influence climate in fundamentally different ways because mantle-derived carbon represents a net transfer from deep reservoirs into the exogenic system, whereas recycled carbon reflects the rapid redistribution among existing reservoirs. Their relative contributions therefore influence both the magnitude of perturbations, isotopic structure and temporal evolution of carbon.

Different components of deep forcing may also exert contrasting climatic effects across different timescales. Carbon emissions generally contribute to long-term warming through greenhouse forcing. In contrast, sulfur emissions as SO_2_ form sulfate aerosols capable of increasing planetary albedo and producing temporary cooling effects. Consequently, climatic responses to magmatic events may exhibit substantial temporal heterogeneity, where persistent warming trends are superimposed by shorter-term cooling episodes. Reduced gases and halogen species further modify atmospheric redox conditions, thereby influencing biogeochemical cycling. Solid-phase inputs provide additional pathways through which deep processes influence climate evolution. Volcanic ash deposition and exposure of fresh igneous rocks enhance chemical weathering rates and nutrient delivery to the ocean [1]. Enhanced weathering promotes atmospheric CO_2_ consumption and further raises nutrient delivery, which can stimulate biological productivity and organic carbon burial over long timescales [7,8]. Therefore, deep forcing should be viewed not as a single forcing variable but as a coupled flux system whose climatic consequences emerge from these combined effects.

The climatic effects of deep forcing are not invariant but depend strongly on the state of the surface system. Identical flux magnitudes can generate substantially different outcomes under different environmental conditions because climate responses are mediated by the structure and organization of surface reservoirs. Surface systems function as dynamic filters that regulate the transmission and amplification of perturbations originating from deep geological processes through attenuation, temporal redistribution, chemical transformation, and nonlinear response. Ocean circulation patterns, continental configuration, weathering efficiency and ocean stratification collectively influence the redistribution of heat, carbon and nutrients across the Earth system. These processes determine whether perturbations are attenuated, amplified or redirected as they propagate through coupled environmental reservoirs. Large igneous provinces illustrate this dependence. The Siberian Traps provide a representative example of how deep-Earth processes can be transformed into global environmental consequences through surface-system interactions [9]. The emplacement of this large igneous province released substantial amounts of CO_2_, SO_2_ and other volatiles through both magmatic degassing and thermogenic processes associated with intrusive activity [10]. However, the magnitude of climatic disruption was amplified by the environmental configuration of the late Permian Earth system. The existence of the Pangea supercontinent restricted ocean circulation and weakened the redistribution of heat and carbon, while stressed marine ecosystems exhibited limited resilience to combined warming, acidification and deoxygenation [11]. Thus, the Siberian Traps event demonstrates that deep forcing alone does not directly determine climatic outcomes; rather, environmental responses emerge from the interaction between geological perturbations and the state-dependent sensitivity of surface reservoirs.

Surface processes therefore influence not merely the magnitude but also the expression of deep forcing. Similar perturbations may generate fundamentally different climate responses because the effectiveness of buffering processes varies across different system states.

Feedback processes regulate interactions between forcing and response and ultimately influence the long-term trajectory of climate evolution. Under moderate perturbations, stabilizing feedbacks may maintain system stability by counteracting environmental change and reducing the persistence of external forcing. The long-term carbon cycle provides a representative example. Increased atmospheric CO_2_ generally enhances temperature and precipitation, accelerating silicate weathering rates and increasing carbon removal from the atmosphere. Operating over geological timescales, this process contributes to climate stabilization and has frequently been invoked as a key regulatory mechanism within the Earth system [7,8,12].

However, feedbacks are not inherently stabilizing. When forcing exceeds the capacity of regulatory processes relative to the background environmental state and response timescales, feedbacks may weaken, reverse, or reorganize, leading to transitions between different climate states. For example, sustained carbon input may exceed the buffering capacity and response timescale of weathering feedbacks, permitting atmospheric CO_2_ accumulation beyond previous equilibrium levels. Importantly, surface environments are not simply receivers of deep forcing but active participants in regulating deep Earth geochemistry [13]. Glacier melting and sea level fluctuations can alter lithospheric stress and deformation localization, thereby affecting mantle melting, magmatic activity, and volatile release [14]. At longer geological time scales, climate-controlled weathering, erosion, and sediment transport processes determine the recycling flux of key components such as carbon, sulfur, and water through subduction zones to the mantle. This deep material recycling significantly modulates mantle metasomatism and continuously regulates the release intensity of deep volatiles. These interactions indicate that the deep and surface systems form a cross layer coupling relationship, and the two are by no means independent ‘forcing’ and ‘responding’ ends.

A major implication of this perspective is that paleoclimate evolution should be interpreted through coupled system dynamics rather than through isolated forcing mechanisms. Importantly, Earth-system coupling operates across multiple timescales, where transient perturbations, intermediate adjustments, and long-term geological processes interact to determine climate trajectories, while background system states regulate the sensitivity to external forcing. Such cross-timescale interactions represent a major challenge for existing Earth system models. Identifying dominant drivers alone may be insufficient for explaining the diversity and complexity of climate behavior observed in geological records. Existing Earth system and carbon cycle models successfully represent many individual processes but often separate short-term and long-term dynamics into distinct parameterizations. Volcanic aerosols are commonly treated as transient perturbations, whereas silicate weathering and tectonic carbon fluxes are represented as independent long-term processes. Although this separation improves computational tractability, it may underestimate interactions operating across timescales.

The future breakthrough lies in building a unified coupled inversion framework that tightly integrates geological reconstruction, process-based analytical models, and data-driven inference [3,15]. Within this framework, geological records provide constraints on key parameters, including deep volatile fluxes, tectonic structural states, surface environmental conditions, and feedback-sensitive boundary conditions. Process-based forward modeling is then used to simulate the propagation of perturbations through coupled climate, carbon-cycle, and biogeochemical systems, thereby testing the consistency between inferred forcing and observed environmental responses. Building upon this physical foundation, data assimilation, state-space methods, and hierarchical models can be employed to reconstruct evolving Earth-system states from sparse geological observations while rigorously propagating uncertainties across multiple archives and temporal scales. AI-assisted analysis further complements these approaches by identifying nonlinear coupling patterns and accelerating parameter estimation, ultimately improving quantitative reconstruction of deep–surface interactions through geological time.

Viewing the Earth system as a coupled nonlinear dynamical system shifts emphasis away from identifying individual drivers and toward understanding how interactions reorganize system behavior. Within this perspective, the central question is no longer simply what forced climate change, but how interactions among Earth-system components determined the trajectory, stability and evolution of climate through geological time.
